# Disk battery ingestion: high clinic risk

**DOI:** 10.1186/1824-7288-41-S2-A27

**Published:** 2015-09-30

**Authors:** Pasquale Di Pietro, Silvia Vignola, Salvatore Renna, Emanuela Piccotti, Arrigo Vittorio Barabino

**Affiliations:** 1 Emergency Medicine, G. Gaslini Istitute, Genoa 16147, Italy; 2 Gastroenterology and Digestive Endoscopy Unit, G. Gaslini Istitute, Genoa 16147, Italy

## 

Over the last ten years disk battery (DB) ingestion have been increasing in children with serious consequences due to the diffusion of lithium battery (LB) that may cause catastrophic damages when lodged in the esophagus.

The severity of injury depends on cell type, size, voltage, location and time of contact with the mucosa because electrical generation of hydroxide ions at the negative pole, leakage of alkaline content in stomach and mechanical pressure.

Because LB are larger (> 20 mm), flatter and have an higher voltage (3V) than alkaline DB (1.5 V) in small children their ingestion increases the risk of esophageal lodgment and tissue damage in just two hours [1,2].

DB ingestion is not witnessed in 92% of fatal outcomes and 56% of major complications; 36% of patients with esophageal lodgment are initially asymptomatic [3]. Clinical presentation can be variable from absence of symptoms to drooling, dysphagia, vomiting, chest pain, or dyspnea, fever, abdominal pain, irritability and feeding refusal and sudden fatal exsanguination for a fistula between esophagus and mediastinic vessels [3,4]. Other complications are trachea-esophageal fistula, laryngeal/esophageal stenosis, esophageal perforation, vocal cord paralysis, tracheomalacia, aspiration pmeumonia, empyema, lung abscess, and spondylodiscitis [2]. Complications can be delayed, as the mucosal lesions may worsen also after DB removal. Plain chest and abdomen X-ray have a primary role to address the diagnosis and locate DB, revealed by the double ring or “halo” effect.

A “sentinel bleed”, isolated hematemesis/melena occurring hours or days before a fatal hemorrage, is another atypical presenting symptom [4]. Exsanguination can occur with the DB still in the GI tract or until 28 days after its removal [1,2].

We propose a new protocol for DB ingestion management in children and stress the necessity of prevention with public awareness campaigns promoted by scientific Societies and preventive information addressed to parents and caregivers [3].

**Figure 1:**
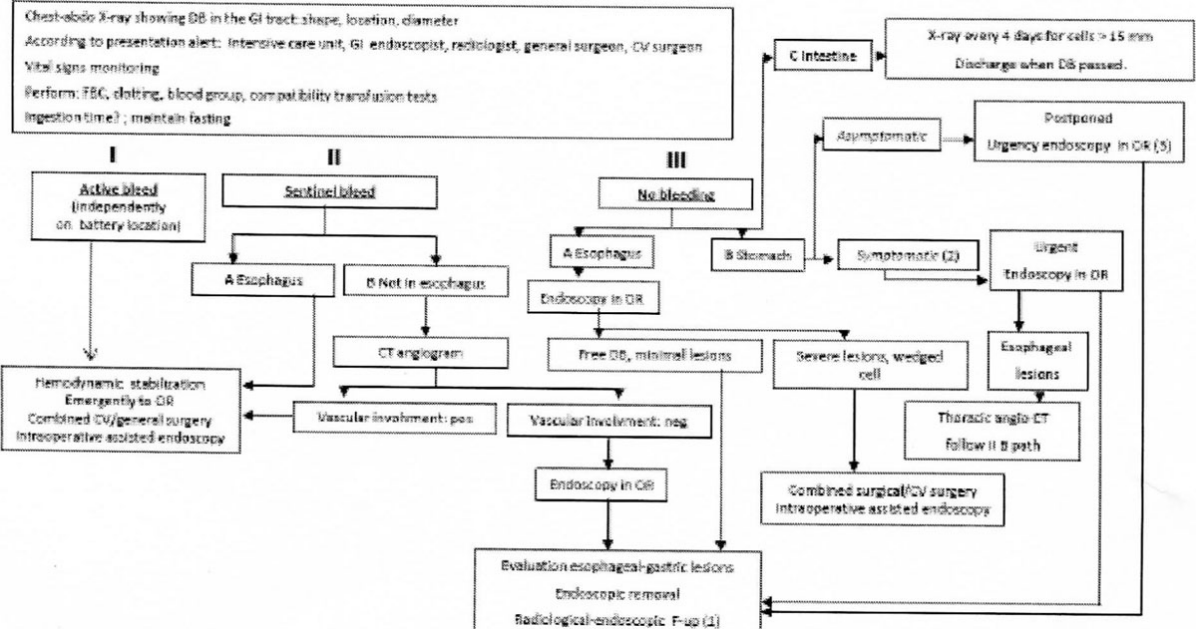
Algorithms for the management of ingested Disk Batteries in children (Lithium Batteries or Alkalike Batteries). 1. The follow-up, above all in case of esophageal lesion, should monitor possible late onset esophageal perforation or vessel fistula. 2. Consider all symptoms, excluding bleeding. 3. Endscopy can be postponed within 48 hrs in not passed cells; reduce the waiting time in case of alkaline battery, very young age or not witness ingestion. DB: disk battery; GI: gastrointestinal, CV: cardio-vasscular; FBC: full blood count; OR: operating room equipped for cardio-vascular surgery; CT angiogram computed tomography angiogram; F-up: follow-up.
